# Systemic Lupus Erythematosus Presented with Bilateral Orbital Edema and Negative Serology

**DOI:** 10.1155/2019/7140534

**Published:** 2019-09-23

**Authors:** Manal Al-Khaldi, Manal Alsabbagh

**Affiliations:** ^1^Registrar, Internal Medicine Department, King Hamad University Hospital, AL-Muharraq, Bahrain; ^2^Registrar, Internal Medicine Department, Dermatology, King Hamad University Hospital, AL-Muharraq, Bahrain

## Abstract

Isolated bilateral periorbital edema with negative serology is an extremely rare presentation of cutaneous lupus erythematosus that may lead to eyelid scarring, infection, or even corneal involvement. The treatment usually comprises a combination of hydroxychloroquine and a tapering dose of systemic steroids. Patients require long-term follow-up as they may develop systemic lupus erythematosus with positive serology later in life. We report a case of a 32-year-old female who presented with chronic bilateral periorbital edema, and the histopathology confirmed cutaneous lupus erythematosus.

## 1. Introduction

Lupus erythematous is a chronic inflammatory autoimmune disease which encompasses a wide range of clinical manifestations affecting multiple organ systems. There are four main types of lupus: neonatal lupus, discoid lupus, drug-induced lupus, and systemic lupus erythematosus [1]. The modified Gilliam grouping system is a modified classification for cutaneous lupus erythematosus which comprises acute cutaneous lupus erythematosus (ACLE), subacute cutaneous lupus erythematosus (SCLE), and chronic cutaneous lupus erythematosus (CLE) [2].

Clinically, each type of cutaneous lupus presents with some specific features. For instance, discoid lupus presents with thick, firm, erythematous, scaly, and well-demarcated plaques that have the potential to heal with scarring, alopecia, and hypopigmentation [3].

The presentation of eyelid edema is extremely rare; the reported incidence is 0.1% as for the presenting manifestation of systemic lupus erythematous with an overall incidence of 4.8% [4]. Diagnosis for these cases is essential to prevent scarring, and long-term follow-up is needed as 5% of the population has the risk of developing systemic lupus erythematosus.

In general, the key characteristic that unites cutaneous lupus erythematosus is the histopathology presentations of the following features: hyperkeratosis, epidermal atrophy, vacuolar interface dermatitis, perivascular, and perifollicular mononuclear cell inflammatory infiltrate, with or without the presence of civatte bodies and follicular plugging [5].

## 2. Case Presentation

A 32-year-old African female presented to the emergency department complaining of persisted periorbital swelling, joint pain, and fever. The periorbital swelling was initially noticed 3 months before her presentation with mild pruritus and no visual disturbance. In addition, she was having intermittent joint pain involving the wrists, fingers, and shoulders. The fever was subjective and not documented with any history of sweating.

On presentation, she also complained of a two-week history of sore throat and difficulty in breathing. Paracetamol was the only medicine the patient was taking to manage her symptoms. She denied using any new medication or product and denied any previous history of allergies.

On her arrival, her vitals were normal and stable. Clinical examination revealed bilateral erythematous periorbital edema with violaceous hue, right cervical lymphadenopathy, and mild friction rub on the lower zone of the right lung. She had tenderness of the wrists, proximal metacarpal joints, and shoulders; movement restriction due to pain was noticed. Cardiovascular, breast, and abdominal examinations were uneventful.

Electrocardiography showed a sinus rhythm with a regular rate and no abnormalities. A chest X-ray showed pleural thickening at the right lung base.

The patient was admitted to the internal medicine department for further investigations. Abdominal ultrasound was normal with no abdominal lymphadenopathy. CT chest with contrast showed right lateral pleurodiaphragmatic adhesions and fibrotic bands.

Laboratory investigations revealed mild lymphopenia, an elevated serum ferritin level, and a mildly elevated erythrocyte sedimentation rate (Tables 1 and 2).

The echocardiogram showed a normal left ventricular cavity size with a preserved ventricular ejection fraction of 55%, and the systolic pulmonary artery pressure was 21 mmHg.

For this case, it was of interest to draw a list of differential diagnosis that includes sarcoidosis, Still's disease, systemic lupus erythematosus, rheumatoid arthritis, amyloidosis, chronic blepharitis, eczema, contact dermatitis, psoriasis, and lymphoma. Still's disease was brought to attention due to an elevated ferritin level; however, there was absence of the classic triad of persistent high-grade fever, joint pain, and a distinctive salmon-colored skin lesion.

**Figure 1:**
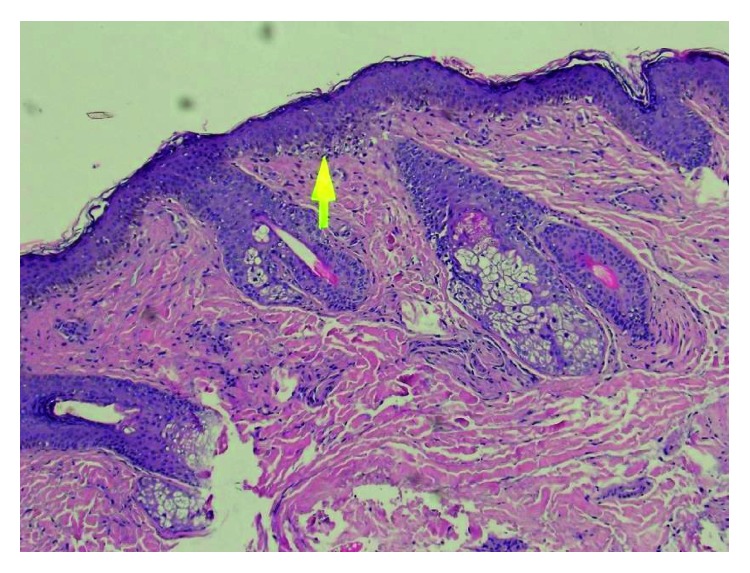
Skin with focal basal cell damage and vacuolar degeneration. There is follicular plugging with a mild dermal inflammatory cell infiltrate. There is no evidence of malignancy. The features are consistent with cutaneous lupus.

Therefore, it remained unclear whether it was systemic lupus erythematosus or amyloidosis until skin biopsy from the eyelid was attempted, and the histopathology results showed degeneration with follicular plugging and dermal inflammatory cell infiltrate. These features were consistent with lupus erythematosus with skin involvement (Figure 1).

The patient was started on prednisolone 40 mg orally once per day with noticeable improvement in periorbital edema and joint tenderness; however, unfortunately, the patient had presented once in the internal medicine clinic after her discharge, while the skin biopsy results were still pending and did not attend further appointments.

## 3. Discussion

Chronic bilateral periorbital edema that poorly responds to topical treatment should raise the suspicion of rare disorders, with possibility of neoplasm or connective tissue disease. Wu et al. reviewed 25 patients presented primarily with periorbital edema with a diagnosis of cutaneous lupus in addition to reviewing 10 cases from the literature. The majority of the patients were females (68%), and only four of the 25 patients had bilateral involvement. Seventy-two percent had their upper eyelid involved. Majority of the patients were misdiagnosed or suspected to have contact dermatitis, dermatomyositis, lymphoproliferative neoplasm, facial cellulitis, and severe angioedema. Our patient was a female with bilateral eyelid erythematous edema. The initial impression was of an allergic reaction and angioedema. Nineteen of the 25 patients as well as our patient had a negative ANA rate in that study (Table 3).

There is a broad implication of shared histological features in lupus erythematosus; 80% of patients had basal cell vacuolar degeneration, 44% had lymphocytic infiltration, and 40% had follicular plugging. Those findings were consistent with our case.

Although our case showed features such as arthritis, pleuritic thickening, and initial lymphopenia, the serologic profile came negative. Wu et al. reported 6 patients, who initially presented with periorbital edema and developed systemic involvement, meeting the American College of Rheumatology criteria for diagnosing systemic lupus. Furthermore, Wu et al. found that 10 cases with lupus erythematosus were reported in the literature with primary presentation of periorbital erythematous edema. Of those, 9 were females, and 6 had a positive ANA. Similarly, cases were misdiagnosed as lymphoproliferative disease, blepharitis, and chalazion.

## 4. Conclusion

Isolated bilateral orbital edema is an extremely rare presentation and found possible to be presented before other skin lesions, the challenges with negative serology impact on diagnosis for cutaneous lupus erythematous, and therefore, these cases are treated as chronic eczema or angioedema. It is essential for patients presenting with eyelid lesions and who fail the treatment for eczema or infections to have a full careful skin examination and skin biopsy for diagnosis.

## Figures and Tables

**Table 1:** Laboratory investigations.

WBC	4.2 × 10^9^/L	ASO quantitative	Negative
Lymphocytes	0.88 × 10^9^/L	Creatine kinase	110.6 u/l
Neutrophil absolute	2.89 × 10^9^/L	Ferritin	1330 ng/ml
Monocyte absolute	0.28 × 10^9^/L	Serum iron	4.46 *μ*mol/L
Eosinophil absolute	0.15 × 10^9^/L	TIBC	34 *μ*mol/L
Basophil absolute	0.03 × 10^9^/L	Transferrin saturation	12.97%
HGB	12.5 g/dl	TFT	TSH 2.387 iu/ml T4 1.09 ng/dl T3 2.86 pn/ml
PLT	162 × 10^9^/L		
Serum creatinine	82 mmol/L	ESR	48 mm/hr
Urea	7 mmol/L	CRP	6.5 mg/L
Na	141 mmol/L		
K	4.6 mmol/L	24 h urine creatinine and volume 1300 ml	9.3 mmol/24 hrs.
Serum albumin	32.5 g/l	24 h protein	0.21 gm/24 hrs.
Total bilirubin	5.57 *μ*mol/l		
ALT	43 u/l	Urinalysis	Glucose 100 mg/dl, protein 30 mg/dl
AST	59 u/l		
GGT	88.2 u/l	Throat swab for beta hemolytic streptococci	Negative

WBC, white blood cell count; HBG, serum hemoglobin; PLT, platelets; Na, serum sodium; K, serum potassium; ALT, alanine transaminase; AST, aspartate aminotransferase; GGT, gamma-glutamyl transferase; ASO, antistreptolysin O; TIBC, total iron-binding capacity; TSH, thyroid-stimulating hormone; TFT, thyroid function test; ESR, erythrocyte sedimentation rate; CRP, C-reactive protein.

**Table 2:** Immunochemistry.

ANA CTD screen	Negative	Anti-RNP70	Negative
Anti-CCP IgG	Negative	Anti-Jo1	Negative
Antirheumatoid factor IgA	Negative	Anti-Scl-70	Negative
Antirheumatoid factor IgM	1.1	Antismith	Negative
Rheumatoid factor quantitative	9.12 iu/ml (<20)	Anti-SS-A/RO, Anti-SSB/LA	Negative
Anticardiolipin IgG, IgM, and IgA	Negative	Anti-U1RNP	Negative
Lupus anticoagulant	Negative	C3	1.5 (normal range?)
Anticentromere protein B	Negative	C4	0.364 (normal range?)
Anti-ds DNA	Negative	Vitamin D	11.5 ng/ml
ACE	Normal range		

ANA: antineutrophil antibody; CTD screen: connective tissue disease screen; anti-ds DNA: anti-double-stranded DNA; ACE: angiotensin-converting enzyme; anti-RNP70 antibody: anti-ribonucleoprotein antibody; anti-Jo1 antibody: myositis-specific autoantibodies directed against the histidyl-tRNA synthetase; anti-Scl-70: anti-topoisomerase antibody; anti-SSA/Ro autoantibodies: anti-Sjögren's-syndrome-related antigen A; anti-U1RNP: anti-ribonucleoprotein.

**Table 3:** A summary of similar presentations that found in different case reports.

Case	Date	Clinical presentation	Laboratory finding	Histopathology	Treatment
A case of discoid lupus erythematosus of the eyelid [6]	2006	A 39-year-old man presented with erosive erythema of the left lower eyelid	Antinuclear antibody, anti-double-stranded (ds) DNA, anti-Sm antibody, anti-SS- A, and anti-SS-B were all negative	A biopsy from the eyelid showed liquefaction and degeneration of the basal layer of the epidermis and the appendage epithelium. The finding is consistent with DLE. The patient response to prednisolone	Prednisolone 10 mg/day, antiallergic drug (cetirizine hydrochloride), and betamethasone sodium phosphate eye drop for 2 years. The skin lesion resolved over 8 months

A case report of lupus erythematosus tumidus converted from discoid lupus erythematosus [7]	2018	A 62-year-old Chinese man presented with a one-year history of recurrent erythematous facial plaques and bilateral swelling of the eyelid	Antinuclear antibodies were positive. Anti-double-stranded DNA antibodies, anti-RO/SS-A, and anti-La/SS-B antibodies were all negative	Histopathology demonstrated liquefaction degeneration of basal cells and perivascular and periadnexal infiltration lymphocytes	Prednisolone 1 mg/kg/day combined with hydroxychloroquine 200 mg twice per day and topical tacrolimus. The patient had complete recovery over 4 months with no relapse over 6 months

Cheek and periorbital peculiar discoid lupus erythematosus: a rare clinical presentation mimicking tinea faciei cutaneous granulomatous disease or blepharitis [8]	2015	A 39-year-old Japanese man was found to have erythema on his right eyelid and right cheek in 2010	Anti-double-stranded DNA antigen, anti- SSA antigen, and anti-SBB antigen were all negative	Histopathology showed parakeratosis and hyperkeratosis of the horny layer and hydropic degeneration and vacuolar changes in the basal layer; the findings were consistent with DLE	Tacrolimus ointment was used with a good response

Discoid lupus erythematosus masquerading as chronic blepharoconjunctivitis [9]	2005	A 33-year-old Caucasian man complaining of his lower eyelid for 8 months was diagnosed with discoid lupus	Antinuclear antibody was negative	Biopsy showed hyperkeratosis of the epithelium, thickened basement membrane, basal cell vacuolation, and dermal inflammation	6 weeks of hydroxychloroquine improved with 6 months follow-up

		A 58-year-old Caucasian woman presented with a complain of eyelid redness and thickening on the right side looks greater	Antinuclear antibody was negative	Biopsy results showed benign chronic inflammation in the dermis, hyperkeratosis of the epithelium, thickened basement membrane, basal cell vacuolation, and telangiectasias, which was consistent with discoid lupus	Treatment with hydroxychloroquine
		A 54-year-old Caucasian woman presented with 25 years of chronic inflammation and scarring of the eyelid and philtrum	Antinuclear antibodies were negative	Biopsy from the right lower eyelid showed epithelial atrophy, focal dyskeratosis, and a thickened basement membrane with an area of focal destruction	Treated with hydroxychloroquine 200 mg twice a day and showed marked improvement over 2 months

		A 41-year-old Caucasian woman presented with 2-year history of eyelid redness and conjunctival infection	Antinuclear antibodies were negative	Biopsy showed granular deposition of IgM, IgG, IgA, and C3 along with the dermo-epidermal junction which was consistent with DLE	Hydroxychloroquine 200 mg was bid orally and showed improvement over 2 weeks of treatment

Discoid lupus erythematosus of the periorbital edema: clinical dilemmas and diagnostic delays [10]	2012	A 50-year-old Caucasian woman presented with a complain of painless and slowly progressive right periorbital swelling	The article did not mention the serology study	Biopsy showed perivascular lymphocytic infiltrate throughout the dermis and the basal epidermal layer, with florid lichenoid changes and vacuolar degeneration which were consistent with DLE	Oral corticosteroid was started, with improvement noticed over 12 months

		A 48-year-old Afro-Caribbean female complains of blepharitis with a demarcated area of depigmentation of both lower lid margins		Biopsy showed chronic inflammation which was consistent with DLE	The patient was treated with hydroxychloroquine with good response

		A 23-year-old Afro-Caribbean male complains of persistent right lower lid swelling		Biopsy showed features that confirmed DLE	The patient was started on hydroxychloroquine, but due to poor compliance there was no response

Eyelid discoid lupus erythematosus and contact dermatitis: a case report [11]	2004	A 71-year-old white female complains of watery eyelids with itching and redness for 10 years	Antinuclear antibodies, anti-DNA, anti-Sm, anti-RNP, anticardiolipin, SS-A (Ro), and SS-B (La) were all negative	Biopsy showed basal cell vacuolar alteration. Showed IgA, IgG, IgM, and C3 intense granular deposits in a band-like pattern along the dermalepidermal junction	Treatment with chloroquine 250 mg/day for 3 months. But this was stopped due to side effects and treatment was changed to corticosteroid

Periorbital edema and erythema: an unusual localization of DLE in a patient with psoriasis [12]	2010	A 33-year-old woman presented with complain of malar erythema and left eye periorbital swelling for 2 years	Antinuclear antibodies and anti-DNA antibodies were negative	Biopsy showed vacuolar degeneration at the basal layer of the epidermis with mild hyperkeratosis. Perivascular lymphocytic infiltration and scattered melanophages on the upper dermis	Hydroxychloroquine 400 mg od po and topical corticosteroid

Severe chronic blepharitis and scarring ectropion associated with discoid lupus erythematosus [13]	2013	A 45-year-old Caucasian woman presented with complain of eyelid redness and irritation for 21 years	Antinuclear antibodies were negative	Biopsy showed hyperkeratosis of the epithelium and a thick basement membrane and a sign of chronic inflammation which was consistent with DLE	The patient was started on hydroxychloroquine 200 mg bid po, with improvement noticed over 2 months
